# A Comprehensive View of the Cancer-Immunity Cycle (CIC) in HPV-Mediated Cervical Cancer and Prospects for Emerging Therapeutic Opportunities

**DOI:** 10.3390/cancers15041333

**Published:** 2023-02-20

**Authors:** Jonathan Peña Avila, Bruno Melo Carvalho, Eliane Campos Coimbra

**Affiliations:** Institute of Biological Sciences, University of Pernambuco (ICB/UPE), Rua Arnóbio Marques, 310, Santo Amaro, Recife 50100-130, PE, Brazil

**Keywords:** cervical cancer, HPV, immunotherapy, CIC, molecular targets, ADC, SCC

## Abstract

**Simple Summary:**

Every year, cervical cancer affects more than 500,000 women worldwide. The persistent infection caused by the human papillomavirus (HPV) is the main risk factor for the development of this type of cancer. Conventional treatments for cervical cancer are often associated with resistance and side effects. Therefore, it is necessary to find new targets for the development of more effective therapeutic approaches. In recent years, an increasing number of studies have been concerned with developing immunotherapeutic strategies for treating cancer. Thus, it is important to investigate new targets, such as the various molecules and cells that are involved in the cancer-immunity cycle (CIC). This process consists of the release of cancer antigens and their destruction by cytotoxic T-cells. Hence, in this review, we discuss the molecular changes that occur at each stage of the CIC for cervical cancer, including the impact of variables such as histological subtype and HPV infection. Moreover, we explore the latest immunotherapeutic approaches that have been adopted, together with their benefits and limitations. In this scenario, current studies are opening up new horizons in clinical practice for a personalized treatment of cervical cancer.

**Abstract:**

Cervical cancer (CC) is the fourth most common cancer in women worldwide, with more than 500,000 new cases each year and a mortality rate of around 55%. Over 80% of these deaths occur in developing countries. The most important risk factor for CC is persistent infection by a sexually transmitted virus, the human papillomavirus (HPV). Conventional treatments to eradicate this type of cancer are accompanied by high rates of resistance and a large number of side effects. Hence, it is crucial to devise novel effective therapeutic strategies. In recent years, an increasing number of studies have aimed to develop immunotherapeutic methods for treating cancer. However, these strategies have not proven to be effective enough to combat CC. This means there is a need to investigate immune molecular targets. An adaptive immune response against cancer has been described in seven key stages or steps defined as the cancer-immunity cycle (CIC). The CIC begins with the release of antigens by tumor cells and ends with their destruction by cytotoxic T-cells. In this paper, we discuss several molecular alterations found in each stage of the CIC of CC. In addition, we analyze the evidence discovered, the molecular mechanisms and their relationship with variables such as histological subtype and HPV infection, as well as their potential impact for adopting novel immunotherapeutic approaches.

## 1. Introduction

Despite the implementation of human papillomavirus (HPV) vaccination programs, cervical cancer remains one of the main causes of women’s morbidity and mortality worldwide [1,2]. Currently, the conventional protocols to eradicate this type of cancer include hysterectomy, cisplatin-based chemotherapy, and pelvic radiation. However, there is a high recurrence rate among treated women that varies between 17% (in cases of the initial stages of the disease) and up to 74% (in advanced stages) [3]. Thus, the identification of new therapeutic targets for devising novel effective therapeutic strategies is vital.

It is believed that immunotherapy against cancer had its onset as early as 1700 when the beneficial effects that some bacterial infections had on cancer were observed [4]. However, it was not until 1891 that the surgeon William B. Coley began to treat patients suffering from inoperable cancers with streptococcal injections and obtained healing and sustained remission in up to 10% of cases [4,5]. Nonetheless, the emergence of chemotherapy and radiotherapy created some skepticism about this type of treatment and the practice was abandoned for a long time. Only in the 1990s did the immunologists James Patrick Allison and Tasuku Honjo resume this idea and they were awarded the Nobel Prize for Medicine in 2018 for identifying the Cytotoxic T-lymphocyte-associated protein (CTLA) 4 and programmed cell death protein (PD) 1, as well as for their role in the negative regulation of the immune response to cancer [6,7]. Since then, a number of different researchers have decided to study the mechanisms involved in the antitumor immune response.

Cytotoxic CD8^+^ T-cells have been identified as the preferred target of study for cancer immunotherapy because when there is a decrease in their activity, this is significantly linked to a failure of the antitumor immune response system [8,9]. However, other T-cell subtypes are also important; for example, CD4^+^ T-cells are crucial because they help to potentiate the cytotoxic activity of CD8^+^ T-cells [10]. Additionally, regulatory T-cells (Tregs), which normally represent only a small percentage of T-cells, have their number increased in cancer and their immunotolerogenic functions have been shown to have a close association with recurrence, tumor progression, and resistance to treatment [11]. Furthermore, the current findings indicate that both innate immunity and adaptive cellular and humoral immunity are required for an effective immune response against cancer [12,13,14,15,16]. Although it is widely recognized that an integrated immune response system is necessary for the development of therapies that lead to the detection and destruction of tumor cells, most research has been focused on studying adaptive cellular immunity (ACI) because of its specificity and immunological memory induction [17,18,19,20,21].

Studies of ACI against cancer have led to the description of the “Cancer-Immunity Cycle” (CIC), which consists of seven key stages beginning from the release of antigens by tumor cells to their destruction by cytotoxic T-cells. Although this process can be divided into stages, it should be noted that it is cyclical, highly integrated, and interdependent. Likewise, as will be addressed later, several molecular mechanisms have been found in each step, which compromise the efficient immune response against cervical cancer, and for this reason, several strategies have been adopted for the development of vaccines or the use of targeted therapies [17,18,19,20,21].

## 2. Molecular Events in the Cancer-Immunity Cycle and Development of Cervical Tumors

**Figure 1:**
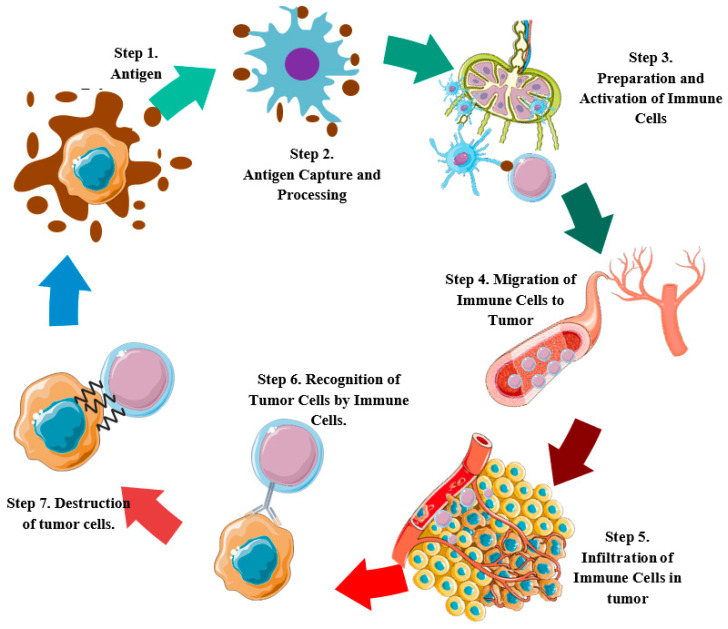
Main processes of the antitumor cellular adaptive immune response system. Step 1, release of antigens by the death of tumor cells. Step 2, recognition and antigen processing. Step 3, priming and activation of T-cells by antigen-presenting cells. Step 4, T-cell migration. Step 5, T-cell infiltration into tumor tissue. Step 6, recognition of tumor cells by T-cells. Step 7, destruction of tumor cells by T-cells.

The CIC consists of seven main phases or stages: during the first stage, there is a release of antigens caused by the death of tumor cells; in the second, antigen recognition and processing takes place; in the third, the antigen-presenting cells prime and activate naive T-cells; in the fourth, the primed T-cells migrate to the tumor; in the fifth, the T-cells infiltrate the tumor tissue; in the sixth, T-cells are recruited, and recognize the tumor cells; and finally, in the seventh step, cytotoxic cells destroy the cancer cells (Figure 1) [17,18,19,20,21].

It has been established that some events that are induced by HPV oncogenes are required for the development of cervical cancer [22]. One such event is the modulation of the host’s immune response to prevent and control infection. At the same time, since HPV allows the panel of immunologic molecules to persist in the epithelium, the virus might give rise to a tumor microenvironment [23]. The tumor microenvironment (TME) is a complex mixture of malignant and nonmalignant cells and surrounding elements that interact with each other to induce cancer progression and the development of the malignant phenotype. The main non-cancerous features of the TME include the extracellular matrix (ECM), blood vessels, immune cells, stromal cells, the presence of oxygen, and nutrient levels. All these factors are favorable to tumors and their progressive growth, and they particularly influence the following situations: (a) when a tumor escapes immune surveillance, (b) the activation of angiogenesis, and (c) cell proliferation, invasion, and metastasis [24]. For instance, the growth of new blood vessels (angiogenesis), which is regarded as a hallmark of cancer, provides the oxygen and nutrients that are required for tumor development [25,26]. In addition, immune cells such as B-cells, T-cells, macrophages, and dendritic cells can alter the behavioral patterns of cancer cells and affect the tumoral immune milieu [27]. The immune system plays a crucial role in the control of a persistent infection of HPV, and the TME can contain immune cells that actively suppress the immune response system by allowing the cancer to evade the immune system [28,29]. The TME in cervical cancer can be characterized as an HPV infection associated with chronic inflammation, which leads to the accumulation of immune cells, such as T-cells and macrophages; this can cause inflammation and oxidative stress in the TME [30,31,32,33,34]. Additionally, the presence of regulatory T-cells (Tregs), tumor-associated macrophages (TAM) of the M2 phenotype, and cancer-associated fibroblasts (CAFs) generates an immunosuppressive TME, which has been attributed to a poor prognosis of various types of cancer [35,36], such as cervical cancer [37,38]. However, a number of studies have demonstrated that factors secreted by cervical cancer cells induced a stable M2 phenotype in macrophages. Macrophages of the M2 phenotype lead to the Th2 immune response, and the development of tumors through the expression of the vascular endothelial growth factor (VEGF) (which suppresses pro-inflammatory Th1 and cytotoxic lymphocyte responses), as well as the transforming growth factor (TGF)-b, indoleamine 2,3-dioxygenase, and programmed death ligand 1 expression [39,40]. Several reports have described the secretion of various factors, including IL-6, IL-13, TGF-b, VEGF, and prostaglandin E2 by CxCa cells [11,17,18,19]. However, it has not yet been clearly established whether the factors secreted by CxCa cells play a key role in the induction or maintenance of the M2 macrophage phenotype. Moreover, fibroblasts and smooth muscle cells, which form a part of the stromal cell structure, interact with the cancer cells and assist in shaping the physical and biological microenvironment of the tumor [41]. In vitro studies have shown that cervical cancer-associated fibroblasts might play a role in the proliferation and survival of tumoral cells [42,43], as well as in the ECM remodeling [44].

Furthermore, it has been found that alterations in the bacterial diversity of the female genital tract occur in CC. These alterations might include an increase in the abundance of certain bacteria, such as Gardnerella vaginalis and Lactobacillus acidophilus and a reduction in the abundance of beneficial bacteria, such as *L. iners*, *L. crispatus*, and *L. taiwanensis* [45]. The exact mechanisms through which these changes in bacterial diversity can lead to the development of cervical cancer are not yet fully understood, but it is believed that the changes in the microbiome might affect the local immune response system and result in the development of precancerous lesions. Further research is needed to fully understand the specific alterations in bacterial diversity and their role in cervical cancer. The different features of the microenvironment may interact and have a reciprocal effect on each other, thus creating an uncertain and evolving ecosystem that contributes to the progression of HPV-related CC. Thus, understanding the TME and its interactions is essential for developing new therapeutic strategies and for improving disease prognosis. As a result, HPV also interferes with the CIC and leads to a more complex process of cancer progression which arises from an interaction between the virus, immune system, and tumor microenvironment.

The following sections will describe the CIC in cervical cancer, from an integrated standpoint by investigating the alterations in each stage and their relationship with the histological subtype of cervical cancer and HPV infection. Additionally, it should be noted that our main focus will be on cervical squamous cell carcinoma (CSCC) and cervical adenocarcinoma (ADC), which are the two main types of cervical cancer that originate from the squamous epithelium and glandular epithelium of the cervix, respectively.

### 2.1. Cell Death and Antigen Release (Step 1)

Several studies have found key changes in the gene expression involved in the mechanisms of programmed cell death or apoptosis in cervical cancer [46]. From an immunological standpoint, this has subsequently led to a decrease in the release of antigens, which are later recognized by the immune system. Some of the key changes include collagen type I alpha 1 (COL1A1) upregulation and phospholysine phospho-histidine inorganic pyrophosphate phosphatase (LHPP) downregulation. This perturbation is related to the reduction in Caspase 3 and BCL2-associated X (Bax) pro-apoptotic proteins and the increase in B-cell lymphoma 2 (Bcl-2) anti-apoptotic protein [47,48,49]. Additionally, the apoptosis-stimulating protein 2 (ASPP2) has been found downregulated in CC cells, decreasing apoptosis by the regulation of autophagy mechanisms [49,50]. Among other alterations inhibiting apoptosis, it has been observed that fibronectin 1 (FN1) impedes the apoptosis of cancer cells and increases their migratory capacity and invasion through the focal protein adhesion kinase (FAK) signaling pathway [51]. Likewise, human hydroxysteroid dehydrogenase-like 2 (HSDL2) upregulation, which participates in fatty acid metabolism regulation, suppresses apoptosis by an unrevealed mechanism in Hela, C33A, and SiHa cells [52,53].

Similarly, there has also been evidence of changes in the expression of long non-coding RNAs (lncRNAs) and microRNAs (miRNAs), as associated with tumor growth stimulation and the inhibition of apoptosis. For instance, lncRNA F-box and leucine-rich repeat protein 19 antisense RNA 1 (FBXL19-AS1) upregulation increase the capture of miR-193a-5p, stimulating COL1A1 expression [48]. This gene is linked to a reduction in the pro-apoptotic Bcl-2 protein. Similarly, the induction of two lncRNAs MEG3 (maternally expressed gene 3) and HAND2-AS1 (heart and neural crest derivatives expressed 2-antisense RNA 1), both downregulated in CC, decreases miR-21-5p expression (upregulated in CC), decreases cell proliferation, and increases the apoptosis of CC cells [54,55,56]. An miRNA that has been found to be downregulated in CC is the miR-433, which is associated with the apoptosis key regulator p53. Then, it was observed that miR-433 upregulation, using in vitro models, upregulates p53 and Bax genes and downregulates murine double minute 2 (MDM2); it also increased the activity of Caspases 3 and 9 and, hence, the apoptosis of tumor cells [57]. Moreover, miR-433 interacts with messenger RNA (mRNA) coding the FAK protein in such a way that the decrease of miR-433 upregulates FAK, PI3K, and p-Akt by inhibiting cell death [57]. Other non-coding RNAs, such as the lncRNA CCAT-1(colon cancer-associated transcript-1 gene), miR-182, and miR-26a-5p have been associated with augmented cell proliferation and reduced apoptosis of CC cells by mechanisms not yet uncovered [52,53,58,59,60]. It has already been showed that the HPV oncoproteins can modulate the expression of protein-coding genes and non-coding RNA genes to promote cervical carcinogenesis [22]. However, it is presently unknown if there is a role of HPV infection in the alterations of those molecules mentioned above, such as the non-coding RNAs. More research is needed to clarify the molecular mechanisms that are involved.

On the other hand, it is well known that E6 and E7 HPV oncoproteins impair the apoptosis process and hence the release of antigens by targeting cellular factors and certain molecules involved in the activation of apoptosis, such as the following: the p53 tumor suppressor protein [61], the tumor necrosis factor (TNF), and the TNF-related apoptosis-inducing ligand (TRAIL) [62]; the miR-21 and the miR-27b, targeting the TNF-alpha and polo-like kinase 2 (PLK2), respectively, to suppress apoptosis [63,64]; and the lncRNA CRNDE, involved in cell survival through the p53 pathway [65,66].

Despite defective apoptosis being associated with the impairment of the immune response because of less available amounts of antigens to be presented to immune cells, this statement needs to be analyzed carefully. Currently, evidence suggests that apoptosis may act as an immunosuppressive or an immunostimulatory signal. Therefore, variables such as stages and the mechanisms involved in apoptosis activation and microenvironment stimuli should be considered [67].

### 2.2. Capture and Antigen Processing (Step 2)

Antigen-presenting cells (APCs) are characterized by having pattern recognition receptors (PRRs) on the outer surface of their membrane, which has the capacity to recognize a wide range of exogenous (pathogen-associated molecular patterns—PAMPs) and endogenous antigens (damage-associated molecular patterns—DAMPs) [68]. Antigen binding with PRRs triggers signaling pathways that allow the internalization, processing, and subsequent presentation to adaptive immune cells (CIC-step 2) [68]. Alterations in these mechanisms have also been observed during the development of CC. Studies have shown that one of the main reasons for the persistence of HPV infection is the ability of the virus to disrupt the receptor and sensory of innate immunity and thus evade the host’s immune system. In light of this, it was found that toll-like Receptor (TLR) 9 plays a key role in the immune response system against HPV infection and cervical neoplasms [69]. In vitro and in vivo investigations showed that the altered expression of TLR9 is associated with the tumor microenvironment in HPV-related cervical cancer [69,70,71,72,73]. Other gene expression studies only found a significant decrease in TLR1 and increase in TLR3 from the epithelial cells in cervical carcinoma [74]. In addition, the presence of the TLR4 receptor has been positively correlated with the hypoxia state of the tumor microenvironment and the expression of the hypoxia-inducible factor-1α (HIF-1α) in CC [75]. Given the high degree of heterogeneity of the alterations observed in PRRs, a possible strategy would be to characterize these changes in a way that corresponds with the different stages and subtypes of CC and thus be able to discern more clearly their functions in the antitumor immune response.

As a result of the interaction between APCs and the tumor microenvironment, the APCs may have their numbers reduced and their functions altered [76,77]. The decline in the number of APCs occurs both from the reduction in the resident APCs and the migratory APCs. At the outset, it should be noted that the infectious cycle of HPV is in itself an evasion mechanism for the host’s immune system because virus replication and the release of novel viral particles do not cause cell lysis, as keratinocyte death is programmed. Thus, there is either a reduction in or complete lack of pro-inflammatory cytokines that activate the migration of APCs [78]. In fact, changes have been found in the expression of some cytokines that stimulate the migration of APCs to the tumor. For example, in CC cells, a reduced migration of APCs to the cervical epithelium for antigen uptake is caused by the downregulation of chemokine C-C motif ligand (CCL) 2, CCL20, and chemokine (C-X-C motif) ligand (CXCL) 14 [79,80,81]. In addition, in vitro and in vivo studies of CC found evidence of a downregulation of CD11b and CD207 markers of LCs, which affected the antigen capture and reduced the differentiation and maturation of dendritic cells (DCs) [79,80,81]. There is evidence to show that the HPV target CCL20 is a cell-attracting chemokine of Langerhans cells (LC), that interferes with the NF-κB pathway [79]. This pathway regulates the expression of multiple genes involved in the pro- and anti-inflammatory processes, such as chemokines, cytokines, and adhesion molecules [82]. Other immune evasion strategies applied by HPV to inhibit the NF-κB pathway include the interaction of viral oncoproteins with the P300/CBP-associated factor (PCAF) and the upregulation of UCHL1 [83,84,85].

Furthermore, studies have shown that HPV oncoproteins interfere with the interaction between the keratinocyte and the LC through the adhesion molecules. Low amounts of LCs arise from the decreased expression of cadherin and the adhesion proteins found in CC [77,86]. However, a recent study that involved the use of in vivo models showed that although the decrease in E-cadherin altered the morphology of LCs, it was not essential to ensure they remained in the epithelium [87]. Additionally, HPV E5 oncoprotein downregulates the cell surface major histocompatibility complex (MHC) class I by interfering with antigen presentation and hence the recognition of HPV-infected cells by the cytotoxic T lymphocytes [88,89]. However, further studies are still needed to explore the changes that are reported in the inside mechanisms of antigen processing in APCs, including factors such as an interaction with different HPV strains, histology features, microenvironment stimuli, and clinical stage, and to determine in what manner this can significantly influence the antitumor immune response.

Genetic variations, such as polymorphisms, can affect different aspects of the immune system, including the capture and processing of antigens. The functional implications of these genetic variations depend on the form and position of the variations in the genome and can range from negligible to substantial changes in the gene function. Bioinformatics analysis in the area of TLR receptors has revealed that polymorphisms can potentially affect the binding sites for transcription factors and disrupt the activation of TLR signaling pathways. However, the functionality inference capability of these tools is limited and additional research is necessary to fully understand the molecular mechanisms behind these changes [90]. Meanwhile, a meta-analysis has been conducted to investigate the impact of prevalent TLR9 and TLR2 gene variations (TLR9 1486 T/C, TLR9 G2848A, TLR2–196 to −174 del/ins) on the incidence of CC. The analysis revealed that the TLR9–1486T/C (rs187084) polymorphism was linked to an increased risk of CC, whereas no link was found with the TLR2–196 to −174 del/ins variant [72,73]. Similarly, another study showed that the IL-12B rs3212227 and TLR9 rs352140 variants were not found to increase the likelihood of CC in any way. Conversely, the XRCC3 RS861539, TNF-α rs1800629, and IL-6 rs1800795 genetic variations were correlated [91]. Another gene polymorphism analysis revealed that the GG genotype of the rs311678 SNP in the cyclic GMP-AMP (cGAMP) gene of the STING (stimulator of interferon genes) pathway was associated with a significantly reduced risk of cervical precancerous lesions. Moreover, the same study also found a significant antagonistic interaction between HPV infection and the rs311678 polymorphism on an additive scale using a three-locus interaction model, involving HPV infection, age range for menarche, and the rs311678 SNP in cGAS [92]. Polymorphisms can also influence the genes responsible for proteasome subunits within MHC-I, potentially influencing antigen presentation. The polymorphism rs2071543 has been found to be associated with an elevated risk of HPV-related CC when present as T/T and T/G genotypes of the proteasome 8 subunit beta and A/A and A/G genotype of the subunit 9 [93].

A recently conducted meta-analysis has explored the relationship between non-coding SNPs and precancerous lesions and CC. The study examined 48 polymorphisms and found 16 SNPs (related to immune proteins, including interleukins, interferon, TLRs, TNF, CTLA, and metalloproteinases), that were closely linked to an increased risk of CC [94]. On this basis, it can be hypothesized that polymorphisms are widely observed in the genes related to the immune response system against HPV-related CC and may represent an evolving adaptation to a dynamic environment. However, the outcomes of polymorphism studies may vary among different populations and ethnicities. There are also some limitations to these studies, including: (a) disparities in the studies included in the meta-analysis, (b) a low number of studies examining certain polymorphisms, and (c) the possibility of publication bias. Nonetheless, conducting an experimental validation of the functional differences between genetic polymorphisms related to HPV-induced carcinogenesis can assist in the development of targeted immunotherapies for specific populations.

### 2.3. Priming and Activation of Immune Cells (Step 3)

After the capture and processing of antigens, the priming and activation of T-cells takes place from naive T-cells (step 3) (Figure 1). This requires the APCs to migrate to the lymph nodes. However, a reduced migratory capacity associated with the low expression of CCR7, a required receptor for T-cell exit from peripheral tissues and entry into lymph nodes, was observed in tumor-infiltrating DCs [95]. Among the mechanisms involved, there was evidence that E6 and E7 proteins from HPV downregulates CCR7 by upregulating IL-6 in cervical cancer cell lines [96,97]. On the other hand, in vitro models showed that LCs from CC that were stimulated by s-Poly-I:C express CCR7 and increases the migration to their ligand (CCL21), which underlines the importance of their expression for the migration of LCs to lymph nodes for priming T-cells [98,99].

Once the APCs infiltrate the lymph nodes, they meet naïve T-cells, where they start their priming and activation. In this phase, it has been observed during in vivo trials of CC that there are LCs prime CD8^+^ T-cells with moderate proliferative activity, a low production of IFN-γ, and high levels of IL-10 [100] and IL-17A [101]. In the specific case of IL-10 (anti-inflammatory cytokine), it should be noted that in CC, keratinocytes, macrophages, and LCs also have an increased production of IL-10 [102], and one study observed that Treg cells, in vitro, through the effects of IL-10, reduces the activation capacity of naive T-cells, which could be interpreted as a feedback mechanism of the immune response [102,103]. It has been demonstrated that the decrease in miR-155 expression levels might be associated with HPV infection and creates a favorable microenvironment for carcinogenesis by decreasing the expression of IFNγ and increasing the expression of IL-10 [104,105]. In addition, in vivo models using K14E7 mice expressing the E7 HPV-16 protein, showed a decrease in the priming of CD8^+^ T-cells by LCs [106], a reduced number of Th1 cells primed by DCs, a predominance of priming T-cells with Foxp3^+^ phenotype, and a high expression of CD73 and folate receptor 4, from CD4^+^ naive T-cells [107]. However, unexpectedly, another study that used an in vivo mice model expressing the E7 HPV-16 protein, despite obtaining a reduction in the number and activation markers of LCs; moreover, the decrease in the priming of CD8^+^ cytotoxic T-cells was independent of the LCs [108]. The latter observation may be related to the fact that there are several subtypes of APCs and these in turn show variable stages of maturation. In support of this, in vitro studies provided evidence that the Langerin^−^ DCs subtype prime CD8^+^ T-cells have an elevated proliferative activity, high production of IFN-γ, and low IL-10 [109]. However, further studies are necessary in which APCs subtypes are directly involved in priming and activating each T-cell subtype, depending on the CC subtype. Thus, for example, when T-cell subtypes were compared in tumor-draining lymph nodes from cervical adenocarcinoma (ADC), there was a greater presence of T-cells, with a predominance of Tregs cells, CD8^+^ T-cells with a higher ‘exhaust’ profile, higher levels of CD8^+^ central memory T-cells (TMC CD27^+^CD45RA^−^), and CD8^+^ effector memory T-cells (TEM CD27^−^CD45RA^−^) than was the case with cervical squamous cell carcinoma (SCC) [110]. Thus, there are differences (from the standpoint of priming and activating T-cells) among the histological subtypes of CC that should be explored.

### 2.4. Migration of Immune Cells to the Tumor (Step 4)

Following this, the primed and activated T-cells by APCs must migrate to the tumor (Step 4). As observed in previous steps, several abnormalities have been found. Thus, the presence of certain chemokines has proved to be especially important. For example, CC cells have increased the production of IL-6, which stimulates stroma fibroblasts to produce CCL20 cytokine through the CCAAT/*enhancer-binding protein β* (C/EBPβ) signaling pathway, which in turn is related to the increased tumor recruitment of CD4/IL17/CCR6^+^ Th17 pro-tumorigenic cells [41,101]. Moreover, in vivo and in vitro models showed that HPV E7 oncoprotein downregulates CXCL14 expression by hypermethylation of the CXCL14 promoter. In this way, the forced expression of this chemokine in CC accelerates the migration of NK cells, CD4^+^ T-cells, and CD8^+^ T-cells to the local environment [111,112]. Likewise, the low expression of XCR1 in DCs has been shown to be important because it reduces the direct migration of CD8^+^ T-cells by the interaction of chemokine Ligand CXCL1, as well as its own activation and that of NK cells [113,114]. Furthermore, in vitro, the cleavage of the chemokine receptor ligands CXCL9, CXCL10, and CXCL11 by the matrix metallopeptidase protein 9 (MMP-9) led to the reduction in T-cell migration; in agreement with the previous findings, the inhibition of MMP9 showed an increased expression of CXCL10, IL-12p70, and IL18 [115].

Some studies have shown that alterations may differ in accordance with the histological subtype of CC. Thus, the migration of T-cells in ADC is lower than in SCC; this singular feature has been associated with a low expression of CXCL9, CXCL10, and CXCL11 in ADC compared with that of SCC and it is believed that this might be attributed to a greater presence of conventional type 1 DCs (cDC1) in SCC rather than in ADC, which is associated with a higher production of cytokines that stimulate the migration of cytotoxic CD8^+^ T-cells to the tumor [110]. Additionally, a low expression of CCL4 and β-Catenin was found, as well as a positive correlation between its levels and tumor-infiltrating cDC1 [110]. Thus, the profile variability of the chemoattraction of T-cell cytokines could partially explain the heterogeneity observed in the migration defects of immune cells in CC.

### 2.5. Infiltration of Immune Cells into the Tumor (Step 5)

After being primed and activated, T-cells infiltrate the tumor tissue (Step 5). In this step, CD4^+^ Th17 T-cells and Foxp3^+^ T-cells have higher infiltration rates than those found in normal tissue. It is believed that these two cell subtypes contribute to tumor progression, through the increase in IL-6, IL-10, and transforming growth factor β (TGF-β) [101,116]. Moreover, one immunohistochemical study has investigated the relationship between the STING levels, CD103+ T-cell infiltration, and a cervical cancer prognosis. This study found that the combination of high STING levels and high CD103+ T-cell infiltration is a means of achieving an improved prognosis in cervical cancer. However, it should be noted that additional research is needed to fully understand the mechanisms underlying these associations and determine the exact potential of STING and CD103+ T-cell infiltration, as therapeutic targets in cervical cancer [117]. Furthermore, the evidence supports the view that changes in the extracellular matrix produced by the development and progression of tumor cells play an important role in regulating the infiltration of immune cells. Thus, for example, tumor cells stimulate the synthesis of matrix components by fibroblasts, by creating denser structures that hinder the infiltration of immune cells such as CD8^+^ T-cells [118]. Among the evidence of mechanisms that facilitate the increase in tumor growth and metastases with reduced T-cell infiltration is the remodeling of the extracellular matrix and stimulus through growth factors and cytokines [115,119]. Accordingly, there has been an increased production of extracellular matrix proteins such as fibronectin 1 (FN1), MMP1, and MMP9 in chronic inflammatory and tumor states of the cervix [51,119,120,121,122]. As well as this, the authors detected a positive correlation between their expression levels and the increase in chronic inflammatory processes, clinical progression, invasion, and tumor metastasis [119,120,122]. For this reason, the combined use of MMP9 with other tumor markers such as CA-125 has been recommended for the diagnosis of CC [121]. In vitro studies with SiHa, HeLa, and C-33A cells were carried out to analyze the migration of these cells and it was found that phenyl-lactic acid (PLA) often produced by microbiota lactobacilli increases cell migration and MMP9 expression [123,124]. It was suggested that there should be an increase in the nuclear translocation of factor IκBα and p65 by PLA stimulus by activating the NF-kB signaling pathway [124]. Among the mechanisms suggested in the regulation of MMP9 expression were an increase in IL-6 production by cervical cancer tumor cells [95].

The process of abnormal neovascularization in cervical cancer demonstrated that it is not only related to the blood supply of tumor cells; it also contributes to establishing a tumor immunosuppressive environment. This is because angiogenesis occurs in an anomalous way and leads to the differentiation of immunosuppressive cells and reduction in the infiltration capacity and function of cytotoxic immune cells [125]. When immunohistochemistry techniques were employed in cervical squamous cell carcinoma, the *hypoxia-inducible factor-1α* (HIF-1α) was (a) found to be upregulated, (b) led to a worse prognosis [126], and (c) was identified as a possible protein involved in this process. It is believed that the hypoxia generated by the tumor microenvironment stimulates the expression of HIF-1α and the vascular endothelial growth factor (VEGF) while causing abnormal angiogenesis and altering the expression of some intercellular adhesion molecules (ICAMs) and vascular cell adhesion molecules (VCAMs) [127].

When different cervical cancer cell lineages infected with different types of HPV were compared, it was observed that depending on the lineage, there are differences in the infiltration of immune cells. For example, in vivo and using nude mice and RAG1^−/−^ models, SiHa (HPV16^+^) and Hela (HPV18^+^) cells showed a greater infiltration of inflammatory cells than C33A cells (HPV^−^) [128]. Moreover, high levels of macrophage migration inhibitory factor (MIF) and CCL5 were observed in all the cells; however, only SiHa and HeLa cells downregulate the expression of ICAM, and the plasminogen activator inhibitor-1 (PAI-1) was upregulated by C33A cells and downregulated by SiHa and HeLa cells [128]. Thus, it is recommended that different types of HPV that chronically infect the cervix should also be taken into account, with the objective of clarifying the differences observed in the infiltration of immune cells in cervical cancer tumors.

### 2.6. Recognition of Tumor Cells by Immune Cells (Step 6)

Subsequently, T-cells infiltrate tumor tissue, and this should allow them to recognize the cancer cells. However, there has been evidence of cervical cancer tumor cells with a downregulation of several genes of the MHC, which results in a reduced recognition of tumor cells by cytotoxic T-cells and NK cells [129,130]. Among the downregulated MHC-I genes that have been found in cervical cancer are human leukocyte antigen (HLA) A, HLA-B, HLA-C, HLA-E, and HLA g [131]. Evidence of head and neck cancer has been provided to identify the downregulation mechanisms involved, as well as the downregulation of CXCL14 expression caused by HPV chronic infection and hence the downregulation of MHC-I genes [112]. It is believed that this may happen in a similar way in cervical cancer [112]. Hence, in vivo and in vitro assays were carried out to provide evidence that circEYA1 a circular RNA is downregulated in cervical adenocarcinoma and has the capacity to capture miR-582-3p upregulating CXCL14 [132]. Additionally, methylome studies, using immortalized keratinocytes, found that the distal promoter element (CGI) of the HLA-E gene shows hypermethylation by E7 HPV oncoprotein and is associated with its downregulation [131]. However, it is necessary to carry out more epigenetic studies with the aim of understanding the specific mechanisms of the immunological recognition of tumor cells by T-cells.

The MHC-II complex genes have also been the target of changes in their expression. Thus, in vivo models, using K14.E7 mice, showed the downregulation of MHC-II in infiltrating epidermal LCs, and the upregulation of genes associated with a decreased immune response, such as Indoelamina-2-3-dioxygenase (IDO) 1, Arginase 1, IL-12/23p40, and IL-6 [106]. Furthermore, it was found in cervical cancer that Foxp3^+^ Treg cells, in vitro upregulation of membrane-associated E3 ubiquitin ligase RING-CH (MARCH) 1, and E3 ubiquitin ligase. These proteins degrade CD86 and MHC-II of DC, which are mediated by the IL-10 produced by Foxp3^+^ Treg cells [103]. The proteins present in the extracellular matrix also seem to play an important role in the recognition mechanisms of tumor cells. In light of this, evidence was found that galectin protein (Gal) 3 prevents the interaction between TCR and CD8 receptors, causing inactivation and inducing immunosuppressive activity [133]. Thus, the recognition of tumor cells by immune cells may be altered globally and not only by the MHC-I complex pathway.

### 2.7. Destruction of Tumor Cells (Step 7)

Finally, after the tumor cells have been recognized by T-cells, mechanisms that lead to the destruction of tumor cells should be triggered (Step 7). The signs of defects that are revealed at this stage include the upregulation of co-inhibitory molecules such as inducible T-cell co-stimulator ligand (ICOSLG), CD276, V-set domain-containing T-cell activation inhibitor (VTCN), and programmed cell death protein ligand (PD-L), which has been shown to be one of the main inhibitory mechanisms of antitumor immune response [134,135]. In HPV-associated cervical cancer, high levels of PD-L1 and interferon-inducible 16 receptor (IFI16) expression have been observed, and this expression has been associated with tumor progression. Furthermore, it has been shown that IFI16 can stimulate the expression of PD-L1 through the STING-TBK1-NF-kB pathway [136]. Studies conducted recently reveal that the T-cells from cervical cancer patients display a heightened expression of PD-1, which leads to a greater production of TGF-β and IL-10, decreased levels of IFN-γ, and impaired T-cell proliferation, thereby establishing immunological tolerance and facilitating tumor growth [135,137]. In corroboration of this, in vitro assays showed that the inhibition of PD-L1 expression in CaSki cells was associated with increased proliferation and cytotoxic activity of CD8^+^ T-cells [138,139]. Additionally, as well as elevated levels of PD-1 expression, CD8^+^ infiltrating T-cells in squamous cell carcinoma have a higher ‘exhaustion’ profile and the upregulation of T-cell immunoglobulin and mucin-domain containing (TIM) 3 and the lymphocyte-activation gene (LAG) 3 [110]. It has been found in in vitro assays that the E2 protein of high-risk HPV downregulate STING, a sensor of the innate immune response system of tumors which assists in the production of type I IFNs [139]. The exact alterations in the STING function in HPV-related CC have not yet been fully elucidated. However, an in vitro study has shown that the HPV16 E7 protein can interact with NLRX1 and increase STING sensor turnover, leading to a reduction in the interferon response and the development and progression of head and neck squamous cell carcinoma (HNSCC). The interaction between the HPV16 E7 protein, NLRX1, and STING found in HNSCC may suggest that there is a similar regulatory mechanism for the host immune response system in CC [140]. In addition, it was reported that the HPV E7 protein might repress the STING expression through epigenetic mechanisms [141]. As a result, it has been suggested that the STING signaling pathway could act as a crucial controller of the CIC, not only by playing a role in destroying tumor cells but also activating DCs, enhancing antigen presentation, activating, and differentiating the growth of T-cells, stimulating the infiltration of cytotoxic T lymphocytes (CTLs) and inhibiting the infiltration of immune-suppressive regulatory T-cells [20].

With regard to CD8^+^ T-cell cytotoxicity, the decreased expression of E-cadherin was found and this was related to a worse prognosis in cervical cancer [142,143]. E-cadherin has been shown to be important for polarization and the release of cytotoxic granules used by CD8^+^ cytotoxic T-cells for killing tumor cells, which are stimulated by the interaction between integrin α_E_ (CD103) β of tumor-infiltrating T-cells and E-cadherin of tumor cells [144,145,146,147,148].

The influence of some metabolites in the cellular immune response has also been studied. Thus, a positive correlation has been found between concentrations of tryptophan metabolites produced by cancer cells and tumor progression and lymph nodal metastasis in cervical squamous cell carcinoma [149]. Among the metabolites studied is quinurenine, while the quinunerin/tryptophan ratio has increased in cervical cancer [150]. An in vitro study using different lineages of cervical cancer showed the upregulation of the tryptophan-degrading enzyme IDO1; however, its downregulation in vitro showed no differences in tumor cell growth [151]. Nevertheless, studies using BALB/c nude and K14.E7 (expressing E7 HPV gene) mice models in vivo showed that the downregulation of IDO1 was significantly associated with an increased number of infiltrating NK cells and decreased tumor growth [151,152]. The mechanisms involved in K14.E7 mice models have been identified by noting that the upregulation of IDO1 is associated with the local secretion of IFNγ and suppression of CD8^+^ T-cells [152].

In this stage, the regulation of the immune response by CD4^+^ T-cells also seems to be particularly important. Evidence shows there is a higher incidence of cervical cancer in patients with immunosuppression by human immunodeficiency virus infection (HIV) [153,154]. It was observed that an increased number of circulating CD4^+^ NKG2D^+^ T-cells and a low expression of CD28 costimulatory receptors in cervical cancer patients is linked to a diminished frequency of cytotoxic markers and reduced production of pro-inflammatory cytokines [155]. Moreover, there was evidence that galectin-1 protein (Gal-1) induces the clearance of CD4^+^ Th17 and Th1 cells and induces Tregs cell proliferation [133].

The interaction between different immune cell subtypes can also influence the destruction of tumor cells. Accordingly, in vitro study models showed that cervical cancer LCs stimulated with s-Poly-I:C have an increased expression of cell maturation markers (CD40, CD80, CD83, CD86, CCR7, MHC1, and MHCII), improved migration, and increased production of pro-inflammatory cytokines related to the stimulation of [cell-mediated cytotoxic response of CD8^+^ cells (IL-1beta, IL-6, IL-12p70, IP-10, TNF-alpha, IFNalfa, MCP-1, MIP-1alfa, MIP-1beta, and RANTES) [99]. However, in vivo experiments using mice have found that the depletion of LCs increases the cytotoxic activity of T-cells [109]. In recent years, it has been observed that CD4^+^ T-cells can be differentiated into a subtype with unclear or controversial functions, such as Th17 cells [156,157]. Studies that analyzed cervical cancer as a whole found that the presence of Th17 cells is associated with a good prognosis [158]. However, analyses involving only ADC showed that the predominance of Th17 cells induces tumor progression [159]. Recently, a higher number of PU.1^+^ T-cells have been found in cervical cancer when compared with normal cervical tissue, but their function is still unknown [160]. Until now, when in vivo models have been employed with CH3 mice, these cells have been shown to have an effective inhibitory function on the growth of oral squamous cell carcinoma, and induced apoptosis by stimulating IL-9 production in tumor cells [161]. However, it is still necessary to clarify their functions in cervical cancer.

Among other mechanisms studied related to kill tumor cells is the metabolism of reactive oxygen species. Thus, in the SCC, there is evidence of an upregulation of dual oxygenase 1 (DUOX1), dual oxygenase 2 (DUOX2), and NADPH oxidase 2 (NOX2) genes. An association has been found with an increase in disease-free survival, probably by mechanisms involving interferon *gamma* (IFN-γ), interferon *alpha* (IFN-α) production, and the activation of NK cell signaling pathways [162]. Table 1 shows each CIC step and their alterations for dysregulated targets in cervical cancer.

## 3. CIC-Targeted Immunotherapies in Cervical Cancer

### 3.1. DNA Vaccines

Based on the alterations found in the release of antigens (Phase 1), there have been studies of alternatives for introducing different molecules that may activate the immune response. Among these, there are therapeutic epitope-based vaccines; however, the impossibility of small peptides producing a strong immune response represents a challenge for the development of these types of vaccines [163]. Hence, some studies have used E6 and E7 HPV epitopes associated with adjuvants, but the results are still in the preliminary stage [164]. Genetic immunization has been documented as an effective strategy for the induction of humoral and cellular immunities in a large number of animal models [165,166]. However, some studies show low immunogenicity, which can be explained by the introduction of genetic material in non-specific cells, and by its inability to replicate or spread through neighboring cells in vivo [167,168]. Thus, there are numerous research studies that are designed to potentiate DNA therapeutic vaccines, such as the optimization of DNA delivery systems to cells, through physical means (electroporation, bio ballistics, tattoo, etc.) and chemical methods (liposomes, nanoparticles, cationic peptides, etc.) [169,170,171,172,173,174,175]. Attempts have been made to improve the gene sequence itself: the codon adaptation for expression in mammals [176]; fusion with other proteins that favor the vaccinal antigen present [177,178,179]; and the incorporation of co-stimulatory molecules such as cytokines and chemokines [180,181,182]. However, the results of these vaccines are still insufficient to allow them to be used as a treatment and they remain in pre-clinical study phases [171].

### 3.2. DCs Based Vaccines

As a result of the identified changes in antigen presentation (Step 2 and 3), therapeutic vaccines using DCs have emerged as a promising treatment option in recent years [183]. The use of DCs for the development of therapeutic vaccines against cancer is based on their capacity to present antigens and induce an effective immune response, through priming and the stimulation of T-cell proliferation and activation [184]. Currently, experiments with therapeutic vaccines against cancer based on DCs involve their exposure to HPV antigens, other types of antigenic proteins, peptides or ex vivo tumor lysate, infection or transfection of DCs with DNA or RNA encoding HPV antigens, and the subsequent delivery of DCs to patients [183,185,186,187]. Some studies have proved to be effective in treating cervical cancer in preclinical stages. For example, DCs pulsed with a fusion protein, formed by the functional peptide of *Mycobacterium tuberculosis* thermal shock protein (MTBHsp70) fused with the extracellular domain of the formyl peptide receptor 1 (FPR1), showed the increasing maturation of DCs, as well as an ability to increase IL-12p70, IL-1β, TNF-α production, and enhance the cytotoxic effects of cytotoxic T-cells (CTLs) in mice [187]. In fact, in a recent study, DC-derived exosomes loaded with peptide E7 and *poly* (I:C) were used, in in vitro and in vivo models, which induced the generation and proliferation of cytotoxic CD8^+^ T-cells, together with an increase in IL-2 and IFN-γ secretion and reduction in IL-10 release [186]. Moreover, in vitro studies, using the biological combination RIX-2 (constituted by: IL-1β, IL-2, IL-6, IL-8, TNFα, GM-CSF, and IFNγ) in LC cells, have shown the upregulation of maturation markers, elevated IL-12p70, CXCL10, and CCL2 production, increased expression of CCR7 and cell migration, as well as an augmented proliferation and activation of cytotoxic CD8^+^ T-cells [188]. However, a number of serious issues need to be clarified to increase the effectiveness of these vaccines and improve the infiltration and retention of effector T-cells, including the identification of membrane receptors and DCs activators, and the determination of which specific subtypes of DCs may be involved in this process for an effective stimulation and activation of T-cells [189].

### 3.3. T-Cell-Based Vaccines

The alterations observed in Steps 4 to 7 of the CIC have given rise to therapeutic strategies that are focused on improving T-cell functions. Thus, there is evidence that E6 and E7 HPV epitope specific CD4^+^ and CD8^+^ T-cells can be produced in vitro, using lymphocytes extracted from lymph nodes of cervical cancer patients [190]. Moreover, phase II clinical trials in patients with metastatic cervical cancer, using the infusion of ex vivo treated T-cells and selected as reactive against E6 and E7; it was observed in some cases that there was a complete or partial regression of cancer [191]. However, currently, an efficacy rate of approximately 30% has been recorded, together with the development of therapeutic resistance [191,192,193]. In addition, a variable response to this form of treatment can be explained both by individual genetic variability and the heterogeneity of cancer cells. In corroboration of this, mutations were observed in interferon gamma 1 and HLA-A receptors [194]. Moreover, there was evidence that T-cell reactivity against HPV16-infected cervical cancer tumors is different from the reactivity observed in HPV18-infected tumors [195]. Thus, before it can be employed to improve the effectiveness of this treatment, the design of this type of therapy requires a more in-depth study of the genetics of each individual and the characteristics of the tumor prior to administering an ex vivo treatment to the T-cells.

### 3.4. Non-Coding RNA-Based Therapies

Studies on resistance to immunotherapy have found that non-coding RNAs can modulate these processes [196]. Among the microRNAs studied in cervical cancer, it was found that the expression of PD-L1 can be downregulated by stimulation with miR-140/142/340/383 and the suppression of miR-18a [197]. This strategy is of great value since the use of anti-PD-1/PD-L1 antibodies in phase I and phase II clinical trials in cervical cancer showed low effectiveness and resistance to treatment [198,199]. Another study showed that the administration of miR-34a and sPD-1, using subcutaneous cationic lipid microbubbles in mice, led to an increased IFN-γ production associated with increased antitumor immune response [200]. Recently, other preclinical studies of cervical cancer have recommended inducing E-cadherin expression by stimulating the expression of miR-185-5p [201], using miR-126 for the induction of cytotoxicity mediated by TNF-α and FasL [202] and using lncRNA HOX (HOTAIR—long *non-coding RNA HOX transcript antisense),* which acts competitively against miR-148a and has demonstrated an ability to participate in the regulation of HLA-G expression [203]. However, the evidence suggests that it is still necessary to improve these strategies.

### 3.5. CRISPR/Cas9 Gene Editing

A new tool to knock out the expression of the HPV oncoproteins, E6 and E7, is the gene-editing tool CRISPR/Cas9. Inturi and Jemth [204] demonstrated that the knockout of these oncoproteins by CRISPR/Cas9 enabled both the p53/p21 and pRb/p21 signaling pathways to be restored, which induced senescence in these cells. With regard to the ability of the CRISPR/Cas system to inhibit cervical tumor cell growth in nude mice, it was observed that the tumor volume was significantly smaller in the mouse that underwent E6/E7 mRNAs’ cleavage by the CRISPR/Cas system than with the control group [205,206]. Another study demonstrated that the disruption of HPV oncogenes by CRISPR/Cas9 in the HPV-positive oropharyngeal squamous cell carcinomas (OPSCC) results in the restoration of the cGAS-STING pathway. This result provides new insights into the treatment required for targeting the HPV infection, as well as cervical cancer, since the STING has been regarded as a potential new immunotherapeutic target for cervical cancer [207,208].

In a recent study which employed SiHa cell-xenografted humanized SCID mice, the blocking of the PD-1 pathway through CRISPR/Cas9 was analyzed together with the knockout of HPV oncogenes. The results demonstrated that the lymphocyte function had been restored with an increased number of CD8^+^ and CD4^+^ T-cells, as well as dendritic cells [209]. Additionally, the study by Zhen and colleagues [210] demonstrated that the delivery of CRISPR/cas9 liposomes, in vivo, can eliminate HPV, which results in an increased number of CD8 T-cells and the expression of pro-inflammatory cytokines. A reduction in the number of T reg cells and myeloid suppressor cells was also observed.

The innovative forms of delivery of the CRISPR/Cas system represent a huge advance since it allows new clinical approaches to be adopted for the treatment of HPV and cervical carcinoma, and its effectiveness has been shown to be remarkable both in vivo and in vitro. The delivery of CRISPR/Cas components, injected intratumorally, showed a notable reduction in the tumor growth rate and tumor, as well as preserving adjacent structures and ensuring that the technology has good safety conditions for normal cells. The systemic delivery of CRISPR/Cas has also been recorded in the literature and has opened up new and potential therapeutic applications that are not restricted to the application site [211].

## 4. Conclusions

Based on the results of the studies mentioned above, clearly, a high degree of complexity is involved when studying the antitumor immune response since there are several deviations in all the phases of the cellular adaptive immune response against cervical cancer [212]. Likewise, cytotoxic CD8^+^ T-cells play a key role in destroying tumor cells. However, evidence suggests that these alone are not enough to achieve an efficient antitumor immune response. Thus, it is necessary to examine the study in conjunction with other cell types involved in CIC. For example, functions of different APC subtypes can influence both the priming and activation of T-cells, as well as their migratory and effector capacity. The immunosuppressive function of Treg cells should also be taken into account because there is a constant and dynamic interaction between the different types of immune cells and tumor cells. Moreover, because many studies conduct cervical cancer analysis in a holistic way, there are some contradictory results between cervical cancer subtypes such as ADC and SCC. Hence, the separation of studies according to their histological subtype could help to clarify the mechanisms involved in the immune response in a more effective way.

Currently, the strategies adopted to combat these alterations are not sufficiently effective and only a small group of patients have benefited [196,213]. This may be yielded by the fact that most of the identified therapeutic targets were the result of reductionist analyses which have contributed significantly to a better understanding of the mechanisms of the immune response. Nevertheless, the immune response is highly integrated and requires therapies that reach multiple targets, while preventing compensation mechanisms from generating a resistance to treatment. Similarly, future therapeutic approaches should have a more personalized view. For instance, differences in the immune response against cervical cancer caused by variables such as histology subtypes, HPV strains, age, and clinical stage should be further explored.

## Figures and Tables

**Table 1:** Potential therapeutic targets in the cervical cancer-immunity cycle. Description of the main changes found in each of the stages in the cervical cancer-immunity cycle.

Steps of CIC	Upregulated Genes	Downregulated Genes	Alterations	References
Cell death and antigen release	COL1A1 FN1 HSDL2 miR-21-5p CCAT-1	LHPP ASPP2 LncMEG3 MiR-433	Decreased apoptosis.	[46,47,48,49,50,51,52,53,54,55,56,57,58,59,60,61,62,63,64,65,66,67]
Capture and antigen processing	TLR3 TLR4	TLR2 CD11B CD207 CCL2 CCL20 CXCL14 E-cadherin	Decreased number of APCs. Decreased differentiation and maturation of APCs.	[68,69,70,71,72,73,74,75,76,77,78,79,80,81,82,83,84,85,86,87,88,89,90,91,92,93,94]
Priming and activation of immune cells	Il-6. Il-10.	CCR7	Increased priming of T-cells Foxp3^+^. Decreased migration of APCs to lymph nodes. Decreased priming of Th1 and CD8^+^ cells.	[95,96,97,98,99,100,101,102,103,104,105,106,107,108,109,110]
Migration of immune cells to tumors	IL-6	CXCL14 CXCL9 CXCL10 CXCL11 CCL4 β-Catenin	The migration of TH17 pro-tumorigenic cells CD4/IL17/CCR6+.	[41,101,111,112,113,114,115]
Infiltration of immune cells in the tumor	IL-6 IL-10 TGF-β Fibronectin 1 MMP9 HIF-1α	STING	Matrix remodeling. Denser matrix. Abnormal neovascularization. Presence of Th17 and Foxp3^+^ cells. Reduced CD103^+^T-cells	[51,101,115,116,117,118,119,120,121,122,123,124,125,126,127,128]
Recognition of tumor cells by immune cells	March1 Ubiquitin Ligase 3 IL-10 Galectin 3	HLA-A HLA-B HLA-C HLA-E HLA-G CXCL14 CircEYA1	Decreased recognition of tumor cells.	[103,106,112,129,130,131,132,133]
Destruction of tumor cells	ICOSLG CD276 VTCN2 PD-L1 TGF-β IL-10 TIM-3 LAG-3 IDO1 Galectin 1 IFI16 NLRX1	IFN-γ E-cadherin	Circulating CD4^+^ NKG2D^+^ T-cells with CD28^+^ decreases. Presence of Tregs cells. Decrease of Th1 cells. CD8^+^ ‘exhausted’ T-cells.	[20,110,133,134,135,136,137,138,139,140,141,142,143,144,145,146,147,148,149,150,151,152,153,154,155,156,157,158,159,160,161,162]
